# Supplementary material for: Patterns of Intron Gain and Loss in Fungi
Source: PLoS Biol. 2004 Nov 30;2(12):e422. doi: 10.1371/journal.pbio.0020422 (PMC532390; doi:10.1371/journal.pbio.0020422)
Supplement: Table S1 — Also available at http://genes.mit.edu/NielsenEtAl/. (4.3 MB ZIP). [file pbio.0020422.st001.zip › NielsenEtAl/html/360.html]

AN5441.1.NCU00475.1.MG05661.1.FG06893.1


```
 CLUSTAL W (1.82) Multiple Sequence Alignments - Introns Inserted


Sequence 1: AN5441.1	155 aa
Sequence 2: FG06893.1	179 aa
Sequence 3: MG05661.1	156 aa
Sequence 4: NCU00475.1	205 aa
Alignment Length: 205 aa
Number Identitical Residues: 122 aa
Alignment Score (without introns) 4941


MG05661.1 	MS~L~VSG-------------------EKSNFQ----------------~----------
NCU00475.1	MS~R~QTNGSSMRRLPNTDGGFKRCVGMEVDMALQLACGRPRMKTSDTA~FDDARLIFDT
FG06893.1 	MS~L0HRN-------------------HEIEFNTIDAARRTALAPPDRQ1ITCA------
AN5441.1  	MS1L~VSG-------------------EKTNFQ----------------~----------
          	**     .                    : ::                            

MG05661.1 	~----F0ILRLLN2TN~VDGKQKIVYALTKIKGVGRRYSNLVVKKADVDLNKR2AGELTS
NCU00475.1	0SNFNH~ILRLLN~TN2VDGKQKVVYSLTKIKGVGRRYSNLVCKKADVDLSKR~AGELTS
FG06893.1 	~-NDVQ~ILRLLN~TN~VDGKQKVMYALTKIKGVGRRYSNLVCKKADVDLNKR2AGELTS
AN5441.1  	~----Y~ILRLLN~TN~VDGKEKIMYALTQVKGVGRRYSNLVCKKADVDLNKR2AGELTT
          	       ****** ** ****:*::*:**::*********** *******.** *****:

MG05661.1 	EELERVVTILQNPTQYKIPS~WFLNRQRDIVDGKDSQTLANGVDSKLRDDLERLKKIRAH
NCU00475.1	EELERIVTIMQNPTAYKIPA0WFLNRQRDIVDGKDSQILANGVDSKLRDDLERLKKIRAH
FG06893.1 	EELERIVTILQNPTQYKIPT~WFINRQRDIVDGKDSHILANGVDSKLREDLERLKKIRAH
AN5441.1  	EELERIVTILQNPTQYKIPT~WFLNRQRDIVDGKDYQVLSNGLDSKYREDLERLKKIRSH
          	*****:***:**** ****: **:*********** : *:**:*** *:*********:*

MG05661.1 	RGLRHYWGLRVRGQHTKTTGRRGRTVGVSKKKGG
NCU00475.1	RGLRHYWGLRVRGQHTKTTGRRGRTVGVSKKKGN
FG06893.1 	RGLRHYWGLRVRGQHTKTTGRRGRTVGVSKKKGG
AN5441.1  	RGLRHYWGLRVRGQHTKTTGRRGRTVGVSKKKG-
          	*********************************
```
